# Endoplasmic reticulum stress-related gene model predicts prognosis and guides therapies in lung adenocarcinoma

**DOI:** 10.1186/s12859-023-05384-z

**Published:** 2023-06-16

**Authors:** Yuqi Song, Jianzun Ma, Linan Fang, Mingbo Tang, Xinliang Gao, Dongshan Zhu, Wei Liu

**Affiliations:** Department of Thoracic Surgery, The First Hospital of Jilin University, Changchun, Jilin People’s Republic of China

**Keywords:** Lung Adenocarcinoma, Endoplasmic Reticulum Stress, Gene Model, Prognosis, Therapy

## Abstract

**Background:**

The prognosis and survival of lung adenocarcinoma (LUAD) patients are still not promising despite recent breakthroughs in treatment. Endoplasmic reticulum stress (ERS) is a self-protective mechanism resulting from an imbalance in quality control of unfolded proteins when cells are stressed, which plays an active role in lung cancer development, but the relationship between ERS and the pathological characteristics and clinical prognosis of LUAD patients remains unclear.

**Methods:**

LASSO and Cox regression were applied based on sequencing information to construct the model, which was validated to be robust. The risk scores of the patients were calculated using the formula provided by the model, and the patients were divided into high and low-risk groups according to the median cut-off of risk scores. Cox regression analysis identifies independent prognostic factors for these patients, and enrichment analysis of prognosis-related genes was also performed. The relationship between risk scores and tumor mutation burden (TMB), cancer stem cell index, and drug sensitivity was explored.

**Results:**

We constructed a 13-gene prognostic model for LUAD patients. Patients in the high-risk group had worse overall survival, lower immune score and ESTIMATE score, higher TMB, higher cancer stem cell index, and higher sensitivity to conventional chemotherapeutic agents. In addition, we constructed a nomogram that predicts 5-year survival in LUAD patients, which helps clinicians to foresee the prognosis from a new perspective.

**Conclusions:**

Our results highlight the association of ERS with LUAD and the potential use of ERS in guiding treatment.

**Supplementary Information:**

The online version contains supplementary material available at 10.1186/s12859-023-05384-z

## Introduction

As one of the most prevalent types of malignant tumors, lung cancer has been ranked among the highest incidence and mortality rates in many parts of the world [1]. Approximately 85% of lung cancers are non-small cell lung cancers (NSCLC) [2]. As the most predominant subtype of NSCLC, lung adenocarcinoma (LUAD) accounts for approximately 50% of the total lung cancers [3]. Despite numerous breakthroughs in treating lung cancer, the 5-year survival rate after lung cancer is determined to be 10–20% in most nations [4]. Although targeted therapies have improved the prognosis of some patients, drug resistance inevitably occurs, so this group of patients has to choose other treatment modalities. Immunotherapy as a highly promising treatment has led to sustained remission and elongated survival in a certain proportion of patients, but 80% of progressive NSCLC patients do not have a response to the now confirmed immunotherapy with a single checkpoint inhibitor [5, 6]. Platinum-based chemotherapy stays the gold standard of treatment for advanced lung cancer patients [7].

Endoplasmic reticulum stress (ERS) is a state induced by increased synthesis of intracellular proteins, accumulation of mal-folded proteins, changes in cytosolic calcium levels, or imbalances in endoplasmic reticulum (ER) redox homeostasis [8]. However, if the stress on the ER is chronic or severe and quality control of protein folding cannot be met, ER activates its stress receptor, the unfolded protein response, which triggers programmed cell death [9]. This might be the pathogenesis of many diseases [10, 11], including type 2 diabetes, neurodegenerative diseases, and atherosclerosis. It was suggested that the ERS signaling pathway also plays an active part in cancer progression [12]. Serving as a self-protective mechanism, ERS is not only engaged in the proliferation of cancer cells in hypoxic environments [13], it also enables lung cancer cells to acquire resistance to  chemotherapy, radiotherapy and targeted therapy [14]. Meanwhile, ERS as a double-edged sword is also closely related to drug-induced apoptosis of lung cancer cells [15-17].

A growing number of studies imply that ERS is tightly associated with the development of LUAD. However, the relationship between ERS and the pathological characteristics and clinical prognosis of LUAD patients is still unknown and needs to be further explored. At this stage, research on the use of specific biomarkers to construct prognostic models for lung cancer is emerging, and bioinformatics plays an important role during the process. Within this context, we constructed a prognostic model of ERS-related genes, combining the risk scores in the model with clinical characteristics (age, sex, staging, etc.) to establish a nomogram, and found its performance in estimating patient survival was excellent. Finally, we investigated the relationship between risk score and immune cell infiltration, cancer stem cell index, and drug sensitivity in the expectation of guiding individualized treatment of LUAD patients.

## Materials and methods

### Acquisition and processing of data

LUAD patients' RNA sequencing files and the corresponding clinicopathological profiles were downloaded from the TCGA database (https://portal.gdc.cancer.gov/) and the GSE72094 dataset in the GEO database (https://www.ncbi.nlm.nih.gov/geo/). Patients with missing overall survival data were excluded. Ultimately, 902 LUAD patients were included in the downstream analysis (504 in TCGA and 398 in the GSE72094 dataset). Fragments Per Kilobase of exon model per Million mapped fragments (FPKM) values were transformed into transcript volume per million (TPM) values in RNAseq transcriptome data, and R package limma [18] and sva [19] were used to perform batch correction and normalization of RNA-seq obtained from the two platforms. ERS-related genes were downloaded from the GeneCards database (https://www.genecards.org), an integrated bioinformatics database that provides detailed information on all genes that are currently annotated and predictable in humans, and genes with relevance scores ≥ 10 were selected for this study. 376 ERS-related genes were obtained and collected in Additional file 1: Table S1.

After excluding data with incomplete information, we randomized altogether 902 patients in the TCGA database (n = 504) and the GSE72094 dataset of the GEO database (n = 398) as a TRAIN set (n = 542) and a TEST set (n = 360). TRAIN set was employed for model construction and the TEST set for validation. Expression data from all patients were used for nomogram construction, immune microenvironment analysis, and subsequent correlation analysis. GSE31210 (n = 226) from the GEO database was used for external validation of the prognostic model. The clinicopathological characteristics of all patients were summarized in Table 1.

**Table 1:** Clinical characteristics of LUAD patients in each dataset

Clinical characteristics	Total	%
TCGA	504	100
Survival status		
Alive	321	63.69
Dead	183	36.31
Age		
< = 65 years old	238	47.22
> 65 years old	256	50.79
Unknown	10	1.99
Gender		
Male	234	46.43
Female	270	53.57
Stage		
I	270	53.57
II	119	23.61
III	81	16.07
IV	26	5.16
Unknown	8	1.59
GSE72094	398	100
Survival status		
Alive	285	71.61
Dead	113	28.39
Age		
< = 65 years old	118	29.65
> 65 years old	280	70.35
Gender		
Male	176	44.22
Female	222	55.78
Stage		
I	254	63.82
II	67	16.83
III	57	14.32
IV	15	3.77
Unknown	5	1.26
GSE31210	226	100
Survival status		
Alive	191	84.51
Dead	35	15.49
Age		
< = 65 years old	176	77.88
> 65 years old	50	22.12
Gender		
Male	105	46.46
Female	121	53.54
Stage		
IA	114	50.44
IB	54	23.89
II	58	25.67

### Development of a prognostic model with ERS-related gene

The ERS-associated genes associated with overall survival were derived from a univariate Cox regression analysis of the combined standardized data from both datasets. Afterward, R package "glmnet"[20] was used to perform LASSO regression analysis on the prognostic data, and the penalty function was optimized using cross-validation. Prognosis-related genes with *p* < 0.05 were eventually included in the multifactorial Cox regression analysis to develop a prognostic prediction model for LUAD patients consisting of ERS-related genes. The formula is as follows:

$$Risk Score=\sum_{i=1}^{n}{Coef}_{i}*{expression value}_{i}$$

where ${Coef}_{i}$ represents the coefficient of each prognostic gene and ${expression value}_{i}$ means the relative expression level of each gene normalized by z-score. Subsequently, all those patients were split into two groups, high and low-risk, depending on the median risk score.

### The risk score and independent prognostic analysis

The "survival" package in R was used to plot Kaplan–Meier survival curves, and Receiver Operating Characteristic (ROC) curves for each group were generated using the "timeROC" package [21] to show the relationship between the sensitivity and specificity of the model. The "pheatmap" package in R was utilized to display the risk score and survival status distribution for each patient, and the R package was also used to plot the prognostic gene expression heat maps.

Cox regression analyzed whether risk score and clinical characteristics were independent prognostic factors for patients with LUAD. The relationship between risk score models and the patient prognosis was validated in stratified analyses.

### Construction and validation of nomogram

Using the "survival" [22] and "rms" [23] packages, a nomogram was constructed in terms of patients' gender, age, pathological stage, and risk score for predicting the overall survival of LUAD patients over 1, 3, and 5 years. The "survminer" and "timeROC" packages were utilized to plot ROC curves and calibration curves for checking the validity and reliability of the nomogram.

### Protein–protein interaction (PPI) network

Using the STRING online platform (https://STRING-db.org/), a PPI network was constructed for 13 ERS-related genes in the model. Medium confidence (0.04) was selected as the minimum required interaction score. The line color signifies the type of interactive evidence. Active interaction sources come from all the options provided by the site, such as text mining, experiments, databases, co‑expression, neighborhood, gene fusion, and co‑occurrence.

### Enrichment analysis

Gene Ontology (GO) and Kyoto Encyclopedia of Genes and Genomes (KEGG) were employed to annotate and functionally analyze prognosis-related genes using the "clusterprofiler" R package [24], with a filter of the adjusted *p*-value (*q*-value) < 0.05. GO enrichment analysis covers three aspects of biology: molecular function, cellular component, and biological processes.

Gene set variation analysis (GSVA) is a non-parametric, unsupervised method for assessing transcriptomic and genomic enrichment [25]. GSVA translates genetic level changes into pathway-level by performing a composite score on the set of genes of interest to determine the bio function of the specimen. In the study, pathway information was obtained from the Molecular Signatures Database (https://www.gsea-msigdb.org/gsea/index.jsp) (VERSION V7.0) and the GSVA algorithm was used to aggregate each gene set for scoring.

### Tumor immune microenvironment analysis

Based on gene expression data, CIBERSORT can be used to assess the abundance of different cell types in mixed cell populations [26]. RNA-seq data from LUAD patients were analyzed using the CIBERSORT algorithm, and the relative proportions of 22 immune infiltrating cells were calculated. Spearman correlation analysis was used to explore the relationship between genes and immune cells.

The ESTIMATE algorithm calculates the stromal score, immune score, and ESTIMATE score of the tumor to reflect the purity of the tumor [27]. The “ggpubr” package was used to draw violin plots to graphically display the differences in each score.

### Correlation analysis

The information on Tumor mutation burden (TMB) in the LUAD cohort was obtained from the TCGA database and analyzed with the "maftools" R package [28]. The number of somatic nonsynonymous point mutations is displayed in a waterfall plot for each individual. Differences in TMB between high and low-risk groups were also compared and presented as a box plot. In addition, cancer stem cell index score calculation files were downloaded from the TCGA database, and the relationship between risk score and cancer stem cell index was evaluated.

### Pharmaceutical sensitivity analysis

Differences in the half-inhibitory concentrations (IC50) of antitumor drugs in high and low-risk groups of LUAD patients were assessed using the "pRophetic" R package [29], according to the largest database of pharmacogenomics and presented in box plots.

### Statistical analysis

Statistical analysis in this study was performed using the R programming language (version 4.1.1). Genes associated with prognosis and independent prognostic factors were identified using Cox regression analysis. The Kaplan–Meier curve and log-ranch test were applied to analyze differences in survival. In all analyses, *P* values < 0.05 were deemed to be statistically significant.

## Results

**Fig. 1:**
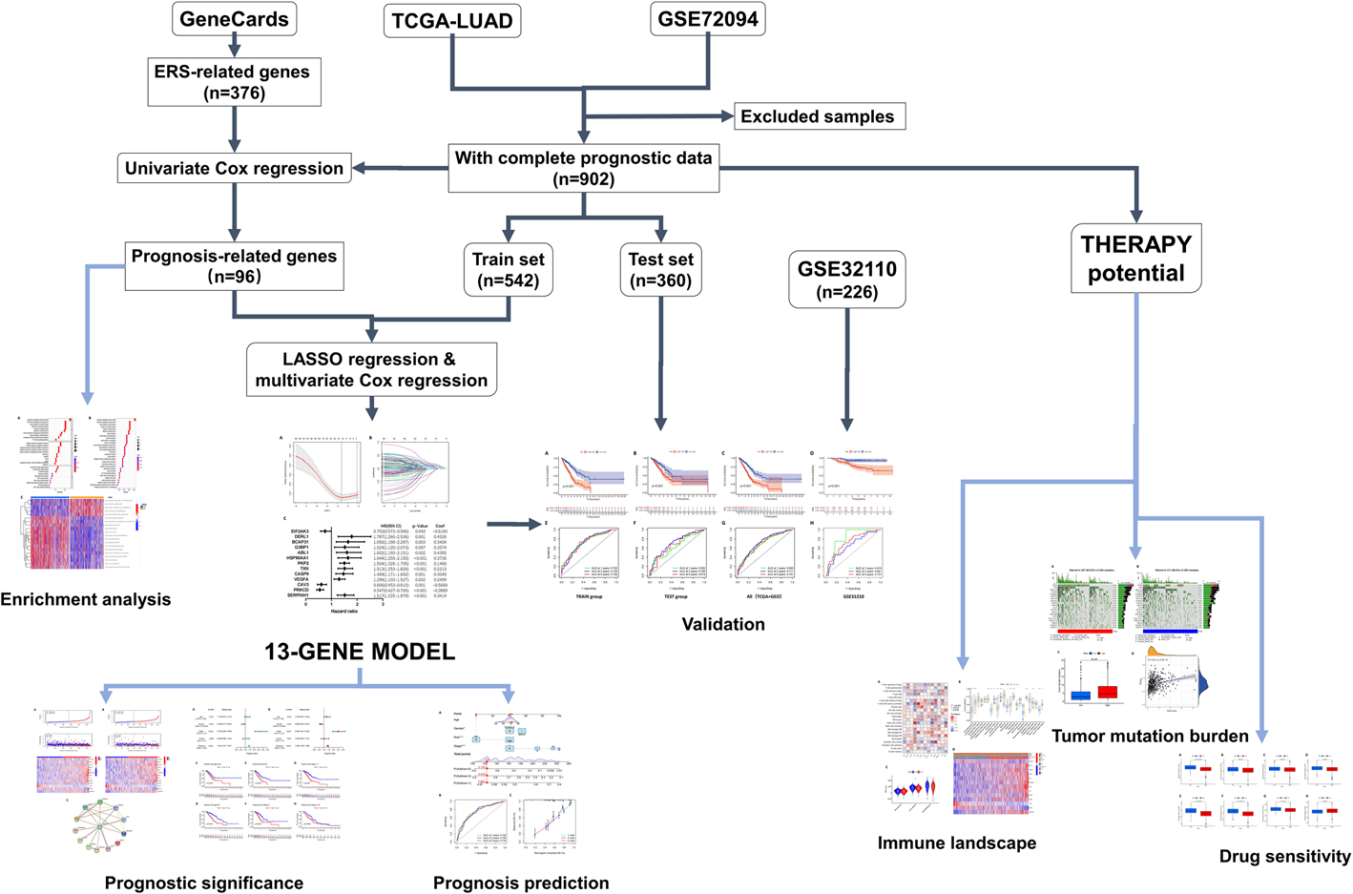
Flowchart of this study

The overall design and flow chart of this study is shown in Fig. 1.

### Prognostic model calculates risk scores to help with patient grouping

**Fig. 2:**
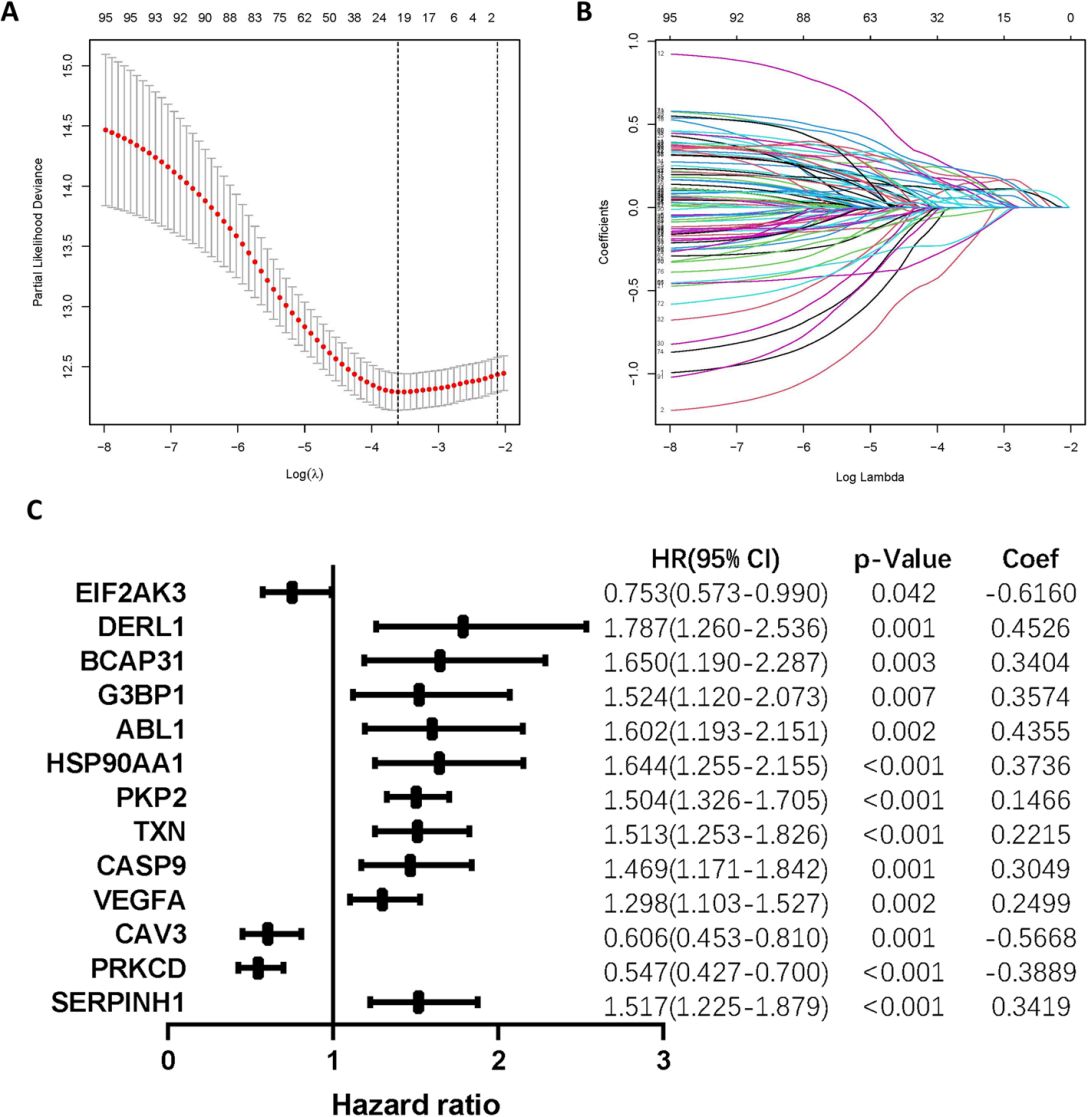
Construction of a prognostic model. **A** The prognostic model is constructed by selecting the variable with the smallest partial likelihood of deviance. **B** tenfold cross-validation for screening variables in LASSO analysis. **C** ERS-related Genes and their correlation coefficients in the prognostic model

By integrating the expression of ERS-related genes with patients' clinical data, we conducted a univariate Cox regression analysis that yielded up to a sum of 96 ERS-associated genes significantly related to overall survival (Additional file 1: Table S2). Following that, LASSO regression analysis was performed in the TRAIN set, and the 21 genes (EIF2AK3, EIF2S1, DERL1, BCAP31, CAT, SLC6A4, G3BP1, HSPA4, ABL1, HSP90AA1, MBTPS2, PKP2, CKAP4, TXN, CASP9, VEGFA, CAV3, PRKCD, SERPINH1, DSP, SLC2A1) corresponding to the lambda.min values were selected and included in the multivariate Cox regression to construct a model including 13 ERS-related genes (Fig. 2A, B), with the hazard ratio (HR), *p*-value, and coefficient of the genes being shown in Fig. 2C. The risk score for LUAD patients can be computed with the following penalized function equation: Risk Score = (− 0.6160 × EIF2AK3 expression) + (0.4526 × DERL1 expression) + (0.3404 × BCAP31 expression) + (0.3574 × G3BP1 expression) + (0.4355 × ABL1 expression) + (0.3736 × HSP90AA1 expression) + (0.1466 × PKP2 expression) + (0.2215 × TXN expression) + (0.3049 × CASP9 expression) + (0.2499 × VEGFA expression) + (− 0.5668 × CAV3 expression) + (-0.3889 × PRKCD expression) + (0.3419 × SERPINH1 expression).

**Fig. 3:**
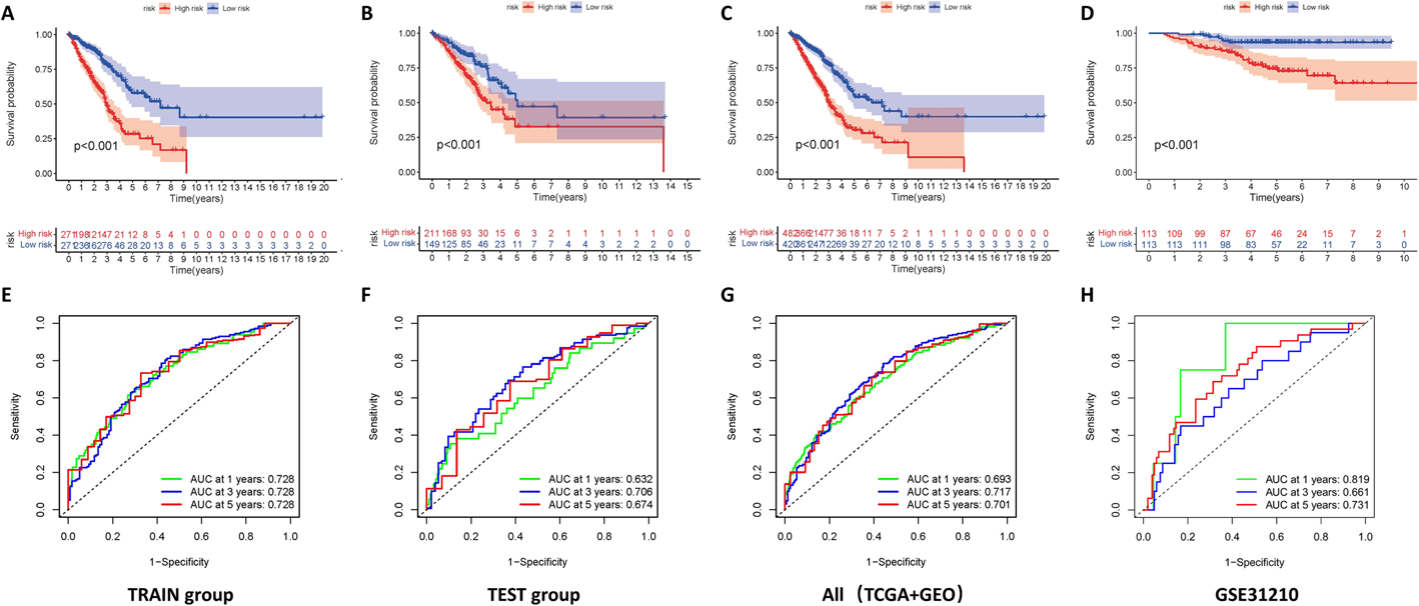
A combination of internal and external validation verifies the predictive performance of the model. Kaplan–Meier curve analysis showed the difference in overall survival between the high and low-risk groups in the TRAIN set (**A**), the TEST set (**B**), and overall patients (**C**). **D** GSE31210 was used as an external validation dataset. **E–H** AUC was obtained from the ROC curve to verify the predictive efficacy of the overall survival time at 1, 3, and 5 years

We separated the entire sample into high and low-risk groups depending on the median risk score and plotted Kaplan–Meier survival curves. The curves showed that the survival rate and survival time were much lower in the high-risk group than in the low-risk group among both TRAIN and TEST sets (Fig. 3A–C, p < 0.001). Time-dependent ROC curves were applied to estimate the model (Fig. 3E–G), with area under the curve (AUC) values reaching 0.728 at 1, 3, and 5 years for the TRAIN set and 0.632, 0.706, and 0.674 at 1, 3, and 5 years for the TEST set. In addition, the GSE31210 dataset was used as an external validation, and the survival curves and ROC curves plotted on its basis as shown in Fig. 3D, H also helped us to validate the good performance of the model for prognosis assessment.

**Fig. 4:**
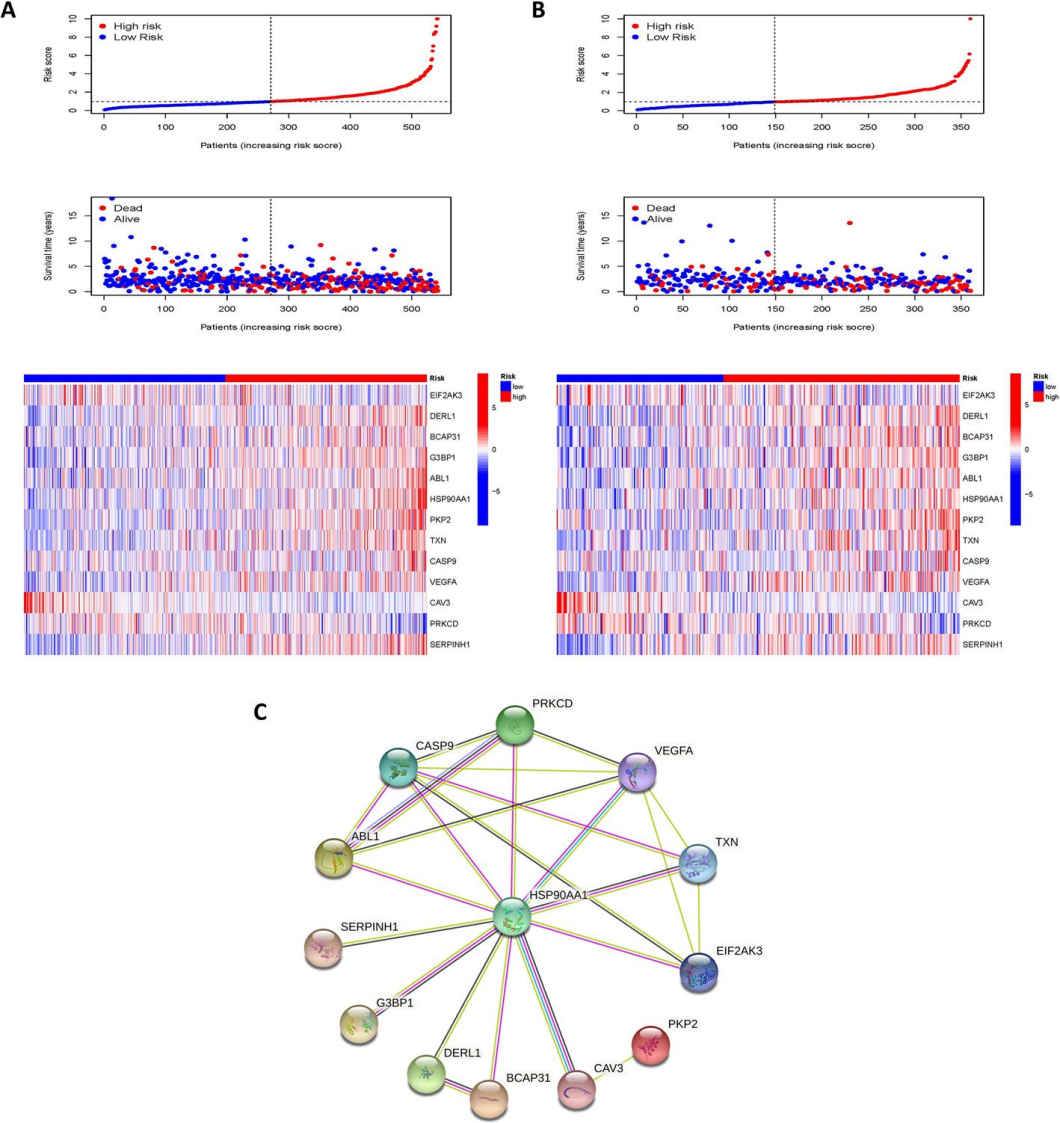
Risk scores distinguish high and low-risk groups. Relationships between risk scores, distribution of survival status, and expression profiles of 13 ERS-related genes in the TRAIN set (**A**) and TEST set (**B**). **C** PPI network of the 13 genes in the model. The minimum required interaction score was set to medium confidence 0.400 and the line color indicates the type of interaction evidence

The trends in risk scores, the distribution of survival status, and the relationship between the 13 genes included in the model and the risk groups are presented in Fig. 4A, B The heat map suggests that high expression of DERL1, BCAP31, G3BP1, ABL1, HSP90AA1, PKP2, TXN, CASP9, VEGFA, and SERPINH1 was associated with high mortality in our high-risk group, while high expression of EIF2AK3, CAV3, and PRKCD was often found in the samples with low-risk scores.

The PPI networks of these 13 genes were mapped to give a better picture of the correlation between the proteins encoded by these genes (Fig. 4C).

### Risk score can be an independent prognostic factor for LUAD patients

**Fig. 5:**
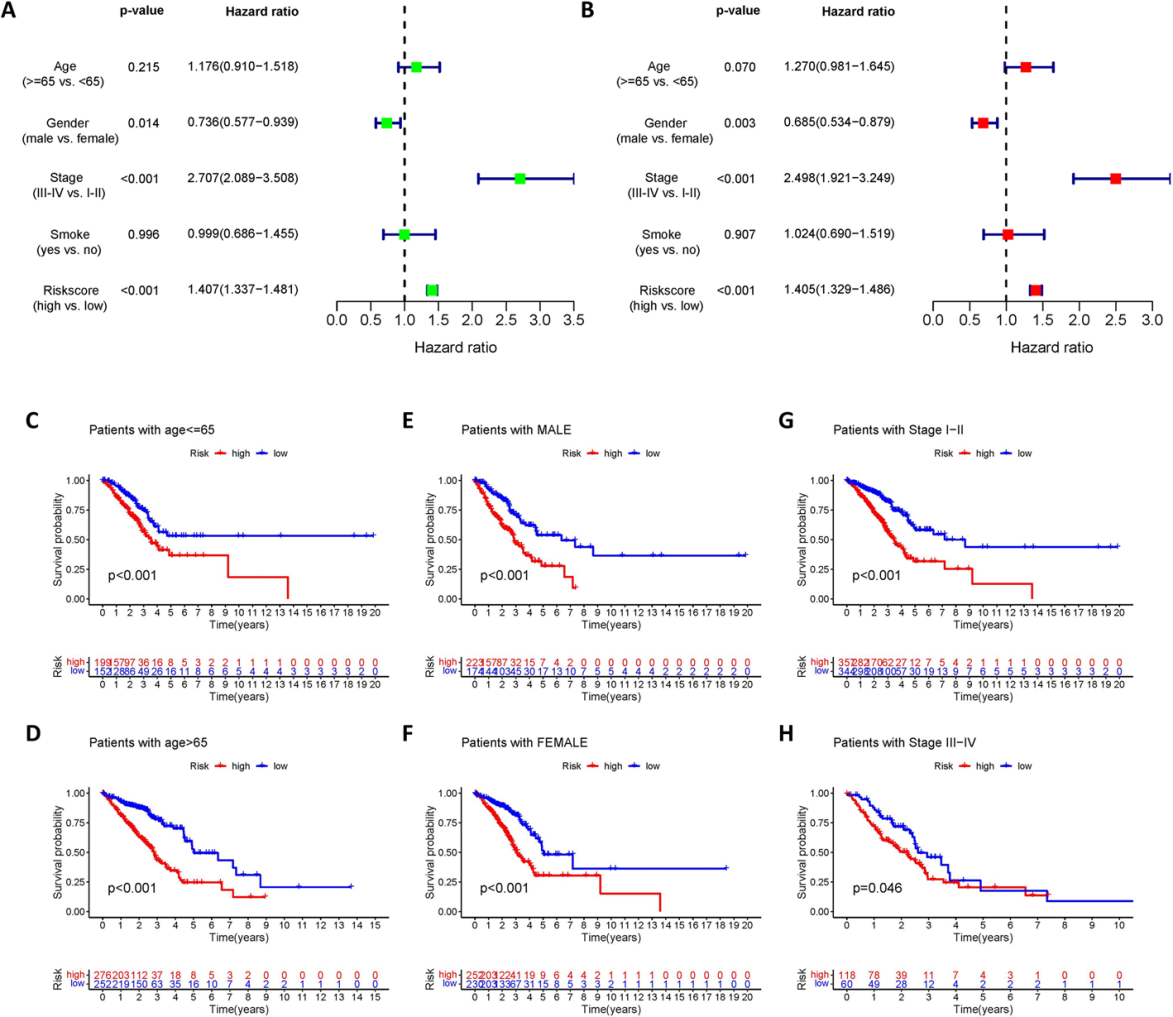
Independent prognostic analysis and stratified analysis. Forest plot of univariate (**A**) and multivariate (**B**) Cox regression analysis based on the clinical information and risk score. Kaplan–Meier survival curves showed significant survival differences between the high and low-risk groups in a clinically stratified analysis including age (**C–D**), sex (**E–F**), and pathological stage (**G–H**)

We performed univariate and multivariate Cox analyses to explore independent prognostic factors in patients with LUAD. Univariate Cox regression analysis revealed that risk scores (HR = 1.407. 95% confidence interval [CI] 1.337–1.481, *p <* 0.001) were significantly related to the prognosis of patients. In addition, gender (HR= 0.736. 95% CI 0.577–0.939, *p* < 0.05), and pathological stage (HR= 2.707. 95% CI 2.089-3.508, *p* < 0.001) were also observed as prognostic factors in patients with LUAD (Fig. 5A). After controlling for confounding variables, multivariate Cox regression analysis disclosed that the 13-gene risk score (HR = 1.405, 95% CI 1.329-1.486, *p* < 0.001) remained an indicator of patient overall survival, and similarly, gender and pathological stage also remained prognostic factors for these patients (Fig. 5B).

We further discussed the applicability of the risk score in the stratified analysis (Fig. 5C–H). Survival analysis suggested marked survival differences between the high and low-risk groups in terms of stratification characteristics including age, gender, and pathological stage. This also validated the robustness of the risk score's ability to predict prognosis.

### Nomogram accurately predict prognosis in LUAD patients

**Fig. 6:**
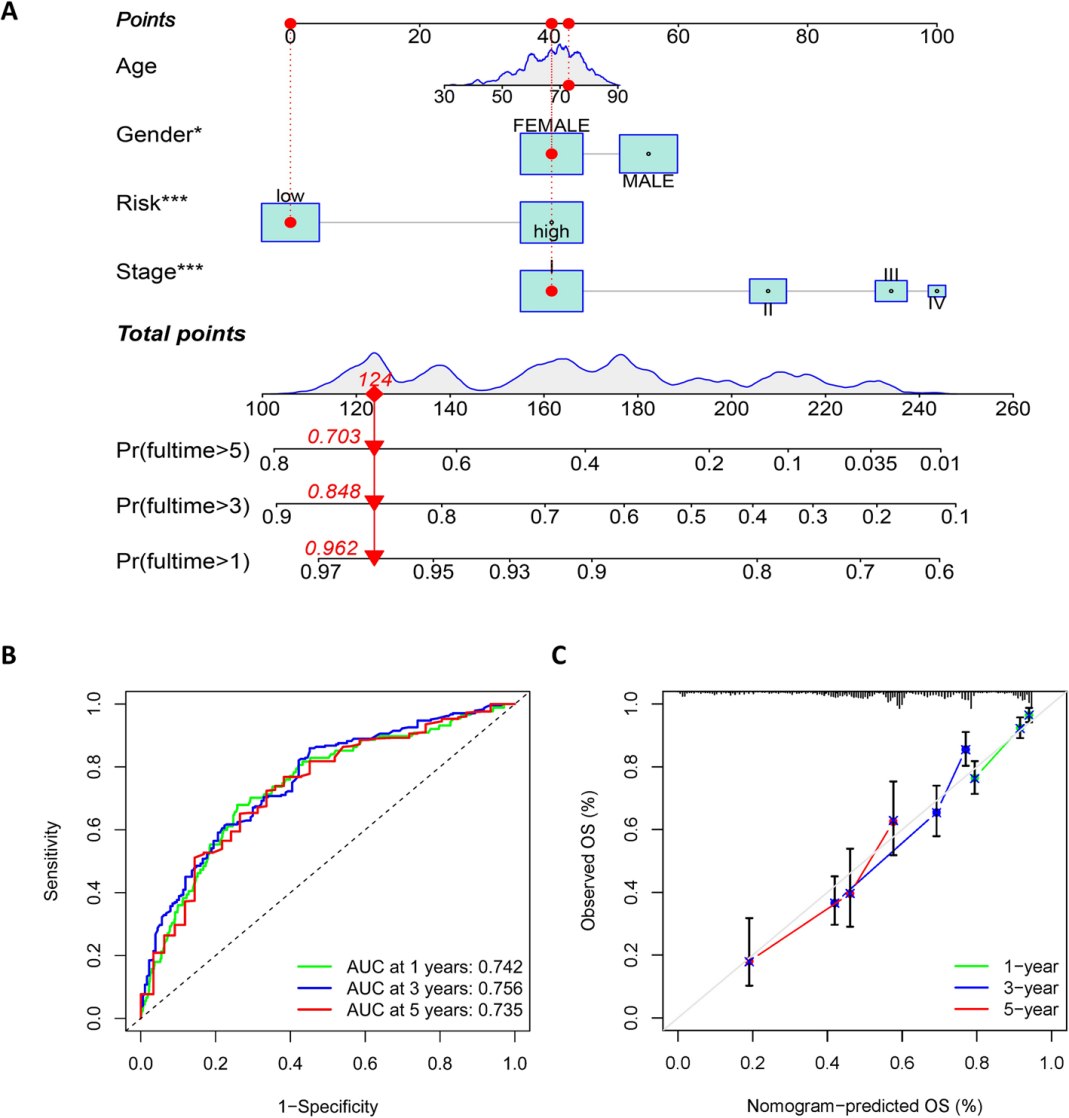
Nomogram is a good tool for predicting prognosis. **A** A nomogram was developed including patient's age, gender, stage, and risk score to predict survival at 1,3,5 year. ROC curves (**B**) and calibration curves (**C**) show that the nomogram has good predictive performance

Based on all sample data obtained, we developed a nomogram including age, sex, stage, and risk score of the model to predict the survival time of patients (Fig. 6A). The nomogram showed that the distribution of different clinical indicators and risk score values contribute differently to the overall scoring process, with the total score broadly forecasting a patient's survival over 1, 3, and 5 years. The ROC curve was used to test the nomogram, and the results showed that the AUC was 0.742, 0.756, and 0.735 for 1, 3, and 5 years, respectively (Fig. 6B), which, combined with the calibration curve demonstrated in Fig. 6C, indicated that the nomogram had excellent predictive power for prognosis.

### Identification of specific signals associated with prognosis

**Fig. 7:**
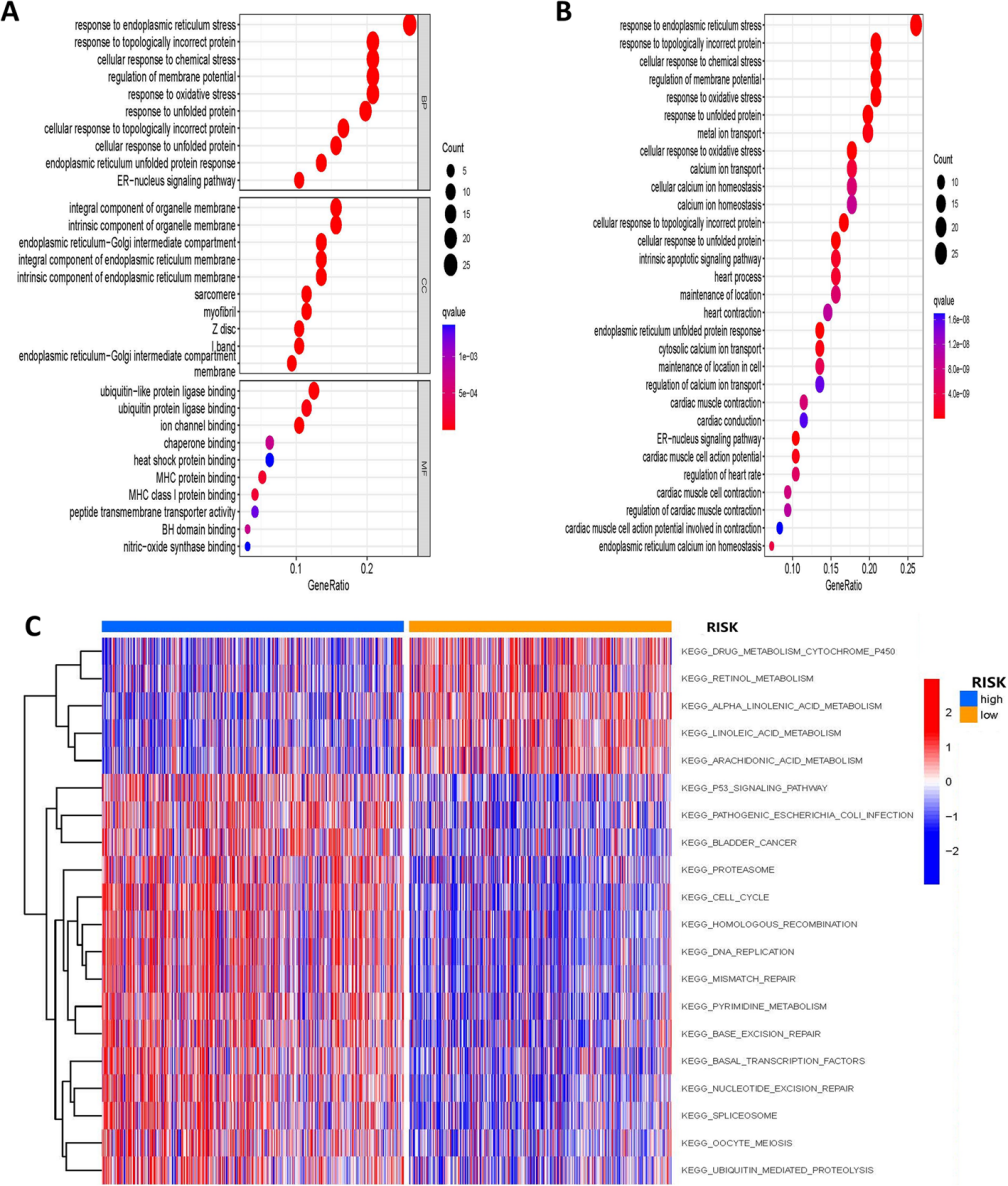
Enrichment analysis of prognostic genes. GO (**A**) and KEGG (**B**) pathway analysis of 96 prognostically relevant ERS-related genes. **C** GSVA analysis reveals differences between specific signaling pathways involved in the two risk groups

We found 96 prognostically relevant ERS-related genes significantly enriched in many pathways through GO and KEGG enrichment analysis. For example, a large number of genes were enriched in response to ERS, topologically incorrect protein, cellular response to chemical stress, etc. in the GO enrichment analysis (Fig. 7A). The enrichment of KEGG (Fig. 7B) displayed that kay genes are primarily engaged in the pathways of response to oxidative stress, response to unfolded protein, and metal ion transport.

Next, we investigated the specific signaling pathways involved in the two risk groups using GSVA analysis and presented them in a heat map (Fig. 7C) and explored the potential molecular mechanisms affecting tumor progression. According to the GSVA results (Additional file 1: Table S3), the differences between the two groups focused on DNA REPLICATION, HOMOLOGOUS RECOMBINATION, CELL CYCLE, MISMATCH REPAIR, and PROTEASOME. These findings suggest that disturbances in the above-mentioned signaling pathways may have an impact on the prognosis of LUAD patients.

### Significant differences in immune cell infiltration between 2 groups

**Fig. 8:**
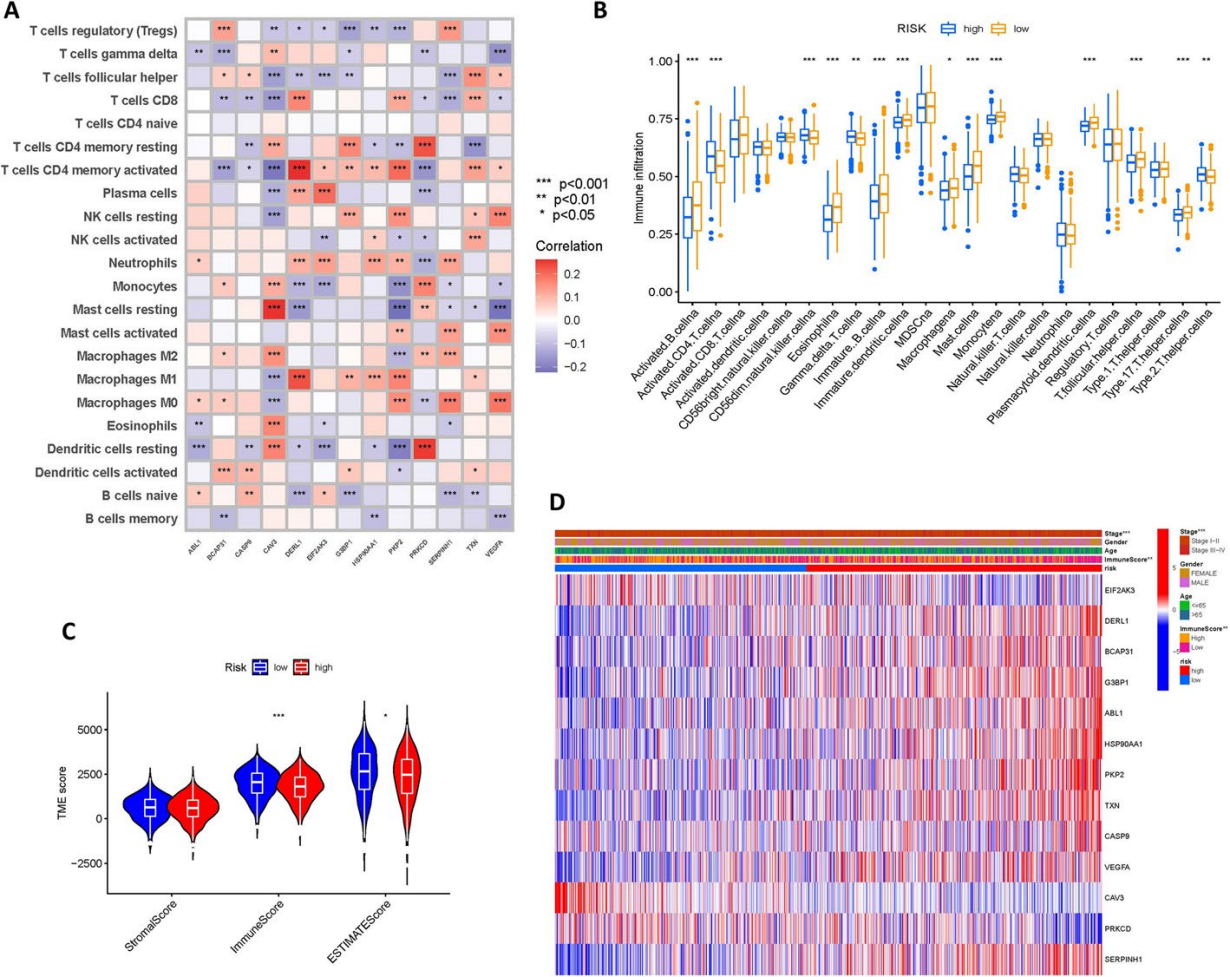
Landscape of immune cell infiltration. **A** Correlation heat map between immune cells and genes in the model. **B** Differences in immune cells between high and low-risk groups. **C** ESTIMATE algorithm was applied to predict the tumor purity and the presence of infiltrating immune/stromal cells in tumor tissues. **D** Heat map of correlations between clinical features, risk score, immune score groups, and genes in the model

It was shown that ERS has a crucial role in immune cells within the tumor microenvironment of LUAD [30]. Therefore, we further investigated whether ERS-related genes affect immune cells. The CIBERSORT algorithm was utilized to unearth the correlations between immune cells and genes in the model and we plotted a heat map to show them (Fig. 8A).

We then analyzed the difference in the content of immune cells in high and low-risk groups. Figure 8B showed that the content of immune cells such as activated B cells, immature B cells, and immature dendritic cells was noticeably lower in the high-risk group, whereas, activated CD4 T cells, CD56dim natural killer cells, and type2 T helper cells were clearly higher.

The tumor microenvironment consists of stromal cells and infiltrating immune cells, reflecting the purity of the tumor. As the number of stromal cells and infiltrating immune cells increases, tumor purity decreases. The results of the ESTIMATE analysis displayed that immune scores and ESTIMATE scores were markedly lower in the high-risk group (Fig. 8C). We further plotted the heat map (Fig. 8D) with the information on age, gender, etc. to better demonstrate the relationship between clinical phenotype, key genes involved in the model, and high and low-risk groups.

### Greater TMB and higher CSC index in high-risk group

Tumor mutation burden (TMB) is simply the total number of mutations carried by tumor cells, specifically the number of somatic mutations per megabyte of the genomic sequence being interrogated. Patients with high TMB have been reported to have higher clinical benefits after immunotherapy, and thus TMB can be considered a potential molecular diagnostic marker for tumor immune checkpoint inhibitors to guide patients receiving treatment [31].

**Fig. 9:**
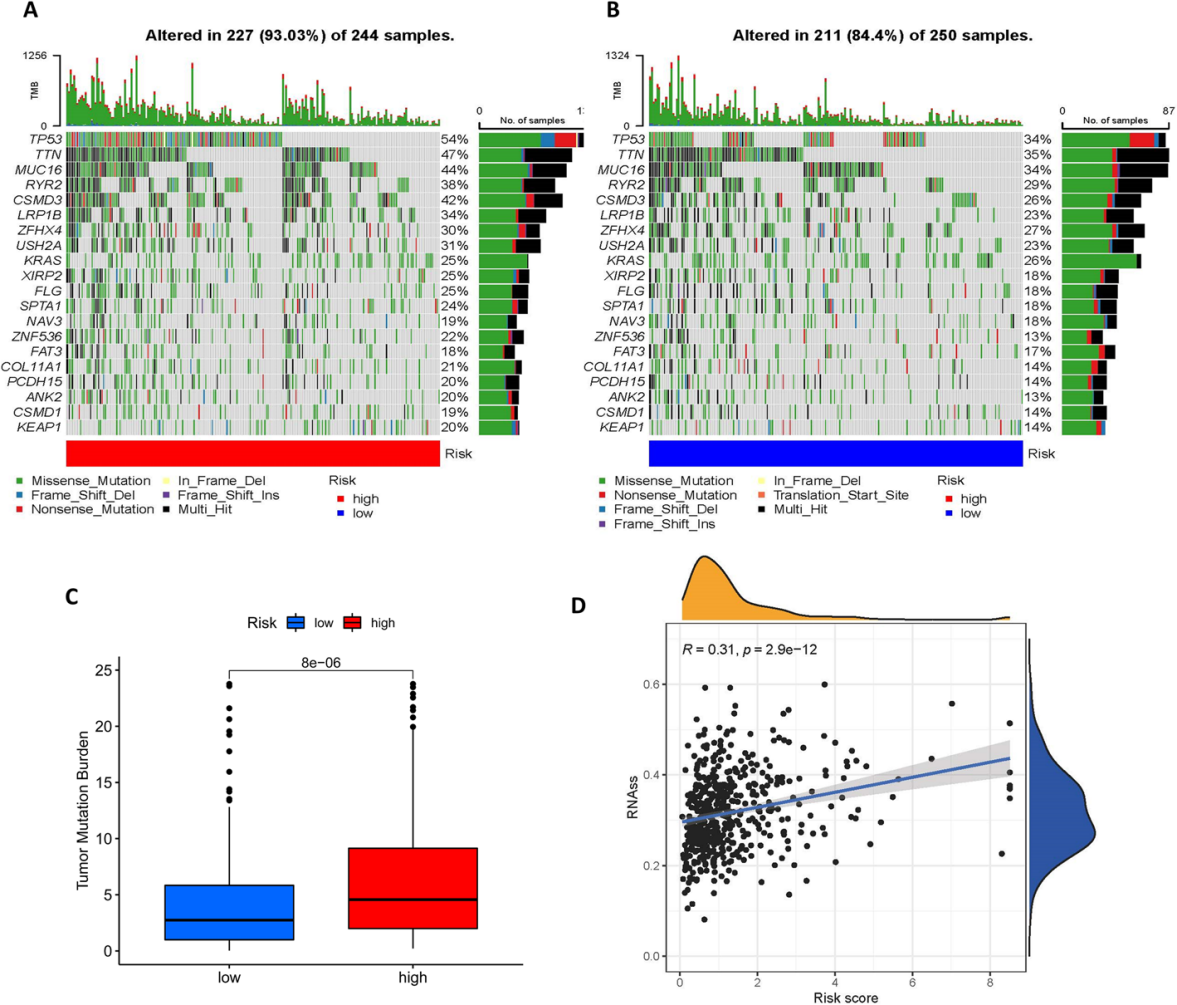
Oncogenic Mutation Landscape and cancer stem cell index. **A**–**B** Waterfall plot of the frequency of mutations in high and low-risk groups. **C** Statistically significant differences in TMB between high and low-risk groups. **D** Risk score is positively correlated with cancer stem cell index

Mutation frequencies for the high and low-risk groups were shown in the waterfall plot Fig. 9A, B, which showed that there is no difference in the names of the top mutated genes in the high and low-risk groups, but the number of mutations differs. We then performed a correlation analysis of TMB (Fig. 9C) and there was a significant difference in TMB between the two groups.

Cancer stem cells (CSCs) are a subpopulation of cells in tumors with the ability to self-renew, which are highly correlated with tumor development and play an important role in other malignant phenotypes such as cancer metastasis and recurrence [32]. Maciej Wiznerowicz et al. generated a cancer stem cell index (mRNAsi) derived from the mRNA expression of 512 LUAD patients using a one-class logistic regression machine-learning algorithm (OCLR) [33]. Spearman analysis reveals a positive correlation between mRNAsi and risk score, with a correlation coefficient of 0.31, *p* < 0.001 (Fig. 9D).

### Predicted differences in drug treatment effects

**Fig. 10:**
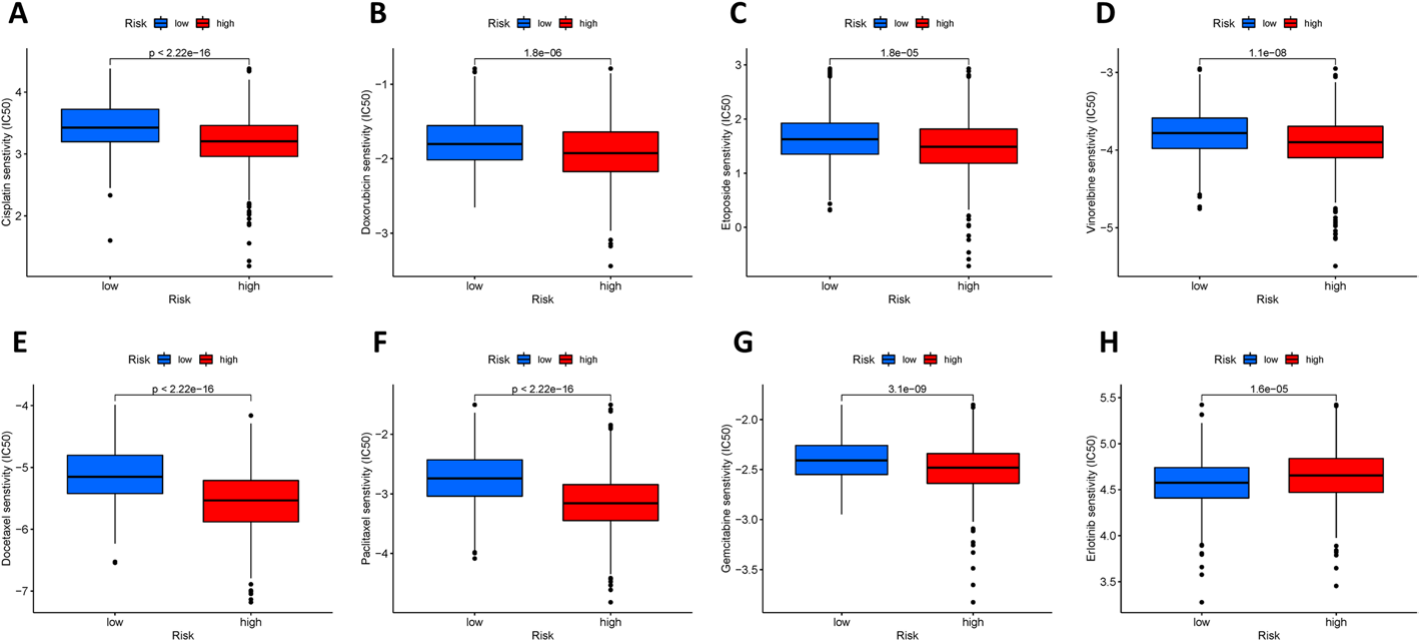
Drug sensitivity analysis. **A–G** The high-risk group is more sensitive to common chemotherapy drugs (cisplatin, paclitaxel, gemcitabine, vinorelbine, docetaxel, doxorubicin, etoposide). **H** The low-risk group has higher sensitivity to the EGFR-TKI erlotinib

Chemotherapy, as a classical lung cancer treatment, is widely used in the adjuvant and neoadjuvant treatment of NSCLC [34]. In this study, the "pRRophetic" R package was used to predict the IC50 of antitumor drugs for each tumor specimen and to further investigate the difference in sensitivity between the two groups. The lower the IC50 value, the more sensitive the drug. From Fig. 10A–G we can see that the common chemotherapeutic agents (cisplatin, paclitaxel, gemcitabine, vinorelbine, docetaxel, doxorubicin, etoposide) are all more sensitive in the high-risk group, while for the erlotinib (Fig. 10H), the sensitivity is significantly higher in the low-risk group, implying that the low-risk group may benefit more from EGFR-TKI therapy.

## Discussion

The endoplasmic reticulum (ER) is an organelle that is essential to all eukaryotic cells and is involved in vital cellular activities. Factors such as hypoxia, hypoglycemia, high temperature, acidosis, calcium levels, redox environment, and energy levels can affect and interfere with the normal function of the ER, leading to ERS and affecting protein folding in the ER lumen [35]. There is growing evidence showing that ERS is an important pathway for cancer cell survival. Due to some characteristics of cancer cells, there is an increased need for protein processing in the ER and the mutant proteins expressed by cancer cells do not fold properly, thereby activating the ERS. The high metabolism of the tumor mass leads to a deterioration of the growth environment such as nutritional deficiencies, and these processes are also potent triggers for the initiation of ERS, resulting in greater tumorigenicity, metastasis, and drug resistance in tumor cells [36]. Interestingly, however, there is sufficient evidence in the literature that the use of inducers to enhance ERS is an effective strategy to induce apoptosis in cancer cells [37-39].

For lung cancer, ERS can induce cellular autophagy in LUAD cells and promote the survival of drug-treated tumors, accelerating drug resistance, recurrence, and malignant development of tumors [40]. In turn, the antitumor activity of some other compounds is achieved through apoptosis mediated with ERS [41, 42], implying that precise modulation of ERS can lead to tumor elimination. Notably, the relationship between ERS and LUAD is not very clear, in which the tumor immune microenvironment may play an important role [43]. So, a thorough comprehension of the mechanisms and results in this field is essential to translate our knowledge into new therapeutic approaches.

In the present study, we applied LASSO regression to construct a 13-gene prognostic model for LUAD patients. Among these genes, high expression of DERL1, BCAP31, G3BP1, ABL1, HSP90AA1, PKP2, TXN, CASP9, VEGFA, and SERPINH1 was highly positively correlated with poorer prognosis. HSP90AA1 (Heat Shock Protein 90 Alpha Family Class A Member 1), as an important node in the PPI network, is a hot topic in the pathogenesis of malignant tumors. Studies have shown that the product of the HSP90AA1 protein, HSP90α, is known to function in the regulation of tumor invasion and migration [44]. In addition, high expression of HSP90AA1 in lung cancer cells is closely associated with lung cancer progression and treatment response [45]. VEGFA (Vascular Endothelial Growth Factor A) is an important regulator of tumor angiogenesis in many solid tumors [46]. Clinical advances in anti-VEGF angiogenesis therapy have been successful and have been translated and utilized in lung cancer [47]. SERPINH1 (Serpin Family H Member 1), also known as HSP47, is an important stress-related protein on the endoplasmic reticulum [48], with higher expression in squamous carcinoma tissues than in normal human bronchial epithelial cells [49]. Similarly, PKP2 (Plakophilin 2) and DERL1 (Derlin 1) are overexpressed in lung cancer cells [50, 51], promote the development of lung cancer, and are associated with lung cancer prognosis [52]. Overexpression of ABL1 (ABL Proto-Oncogene 1) gene is associated with shorter survival in LUAD patients, and animal studies suggest that its inhibitors are effective in treating metastatic NSCLC [53]. G3BP1 (G3BP Stress Granule Assembly Factor 1), BCAP31 (B Cell Receptor Associated Protein 31), CASP9 (Caspase 9), and TXN (Thioredoxin) are involved in multiple biological processes in tumors by regulating signaling pathways [54], thereby promoting tumor cell proliferation and metastasis as well as regulating apoptosis [55]. All of them may be potential targets for tumor therapy [56] and provide new strategies for the treatment of LUAD [57].

For the remaining three genes with Coef less than 0 in the risk score, EIF2AK3 (Eukaryotic Translation Initiation Factor 2 Alpha Kinase 3), which encodes a membrane protein, silencing in cancer cells prevents ERS and induces apoptosis [58]. PRKCD (Protein Kinase C Delta) genes play a key role in growth inhibition, differentiation, apoptosis, and tumor suppression [59, 60]. CAV3 (Caveolin 3) mainly encodes a protein of caveolin-3, which is mainly distributed in the membranes surrounding myocytes, and its dysfunction is associated with many diseases, including cancer and diabetes [61, 62]. Notably, the interrelationship of some genes with LUAD has not received much attention from researchers previously. These genes were identified and given significance in our study and remain to be further investigated in the future.

In the tumor microenvironment, ERS can serve as a novel biological marker [63]. Tumor cells themselves are in a variety of metabolic abnormalities that disrupt the homeostasis of intracellular protein folding and thus induce ERS in cells. This simultaneously induces changes in the microenvironment, such as nutrient deficiency, hypoxia, and imbalance of redox reactions, triggering the occurrence of ERS within the infiltrating immune cells and affecting tumor immunity. For example, ERS affects the myeloid cells in suppressing antitumor immune surveillance [30], and ERS in cancer cells influences the recruitment and function of immune cells, modulates T cell-mediated immune responses, and NK cell-mediated tumor recognition [63], but the exact mechanisms are not clear. Our results showed that the content of multiple immune cells was significantly different in high and low-risk groups, which may be due to disparities in the tumor microenvironment. Moreover, there is also a strong correlation between the genes included in the model and multiple immune cells, which provides clues to understanding the mechanism of ERS involvement in tumor immunity in the LUAD microenvironment. In addition, the TMB of LUAD patients increases with increasing risk scores, and studies have shown that patients with high TMB possess higher survival rates after receiving immunotherapy [64]. This makes the application of our model to immunotherapy possible, and its in-depth exploration will help guide the subsequent eradication of the tumor itself and improve the efficacy of immunotherapy.

CSCs are intimately tied to tumor heterogeneity, antitumor resistance, and metastasis of lung cancer. A significant negative correlation between CSCs and antitumor immunity has been observed [65]. In our results, the cancer stemness index was higher in the high-risk group, but its immune score was lower. This implied the possible existence of genes or pathways that maintain the stemness of tumor cells and inhibit the function of immune cells, thus regulating tumor progression [66]. Importantly, we should be aware that future research on CSC-targeted tumor therapy should be strengthened to enrich the means of tumor treatment [67].

To provide more accurate treatment options for LUAD patients, we used the R package "pRRophetic" to predict the differences in IC50 values of common antitumor drugs between the two groups on the basis of drug sensitivity data. According to the results, chemotherapy, one of the most important treatment modalities for LUAD, continues to benefit patients in the high-risk group (all common chemotherapeutic agents have a lower IC50), while the low-risk group has more treatment options, including targeted therapies (lower IC50 of Erlotinib). Unfortunately, information on immune checkpoint inhibitors is missing here, and our approach was not successful in predicting the sensitivity to immunotherapy in high and low-risk groups.

Of course, our study has some shortcomings. First, due to the limited data on LUAD patients in the public database, the sample size covered in this study was relatively insufficient, resulting in some results not representing the whole picture. Second, the dataset obtained from the public database lacks some important information, which limits us to more in-depth analysis. Furthermore, the 13 genes obtained in the prognosis-related model also need to be validated externally at various levels (genes, proteins, in vivo experiments) to explore and reveal their specific mechanisms.

In summary, we constructed an ERS-related prognostic model and performed drug sensitivity analysis. Our results provide important clues to study the role of ERS in LUAD and are expected to help clinicians understand the relationship between ERS and patient prognosis from a novel perspective. We also expect that it can guide more appropriate drug treatment options for patients in the future.

## Supplementary Information

**Additional file 1**. Supplementary tables for details about ERS-related genes and GSVA analysis results.

## Data Availability

The datasets analyzed in the current study are available from the TCGA database [https://portal.gdc.cancer.gov/] and GEO database [https://www.ncbi.nlm.nih.gov/geo/query/acc.cgi?acc=GSE72094] repository.
